# Use of atypical antipsychotics in the treatment of schizophrenia in the Brazilian National Health System: a cohort study, 2008-2017

**DOI:** 10.1590/S2237-96222023000300015

**Published:** 2023-03-20

**Authors:** Izabela Fulone, Marcus Tolentino Silva, Luciane Cruz Lopes

**Affiliations:** 1 Universidade de Sorocaba, Departamento de Pós-Graduação em Ciências Farmacêuticas, Sorocaba, SP, Brazil

**Keywords:** Antipsychotics, Brazilian National Health System, Schizophrenia, Brazil, Off-Label Use, Cohort Studies, Antipsicóticos, Sistema Único de Salud, Esquizofrenia, Uso Fuera de lo Indicado, Estudios de Cohortes, Antipsicóticos, Sistema Único de Saúde, Esquizofrenia, Brasil, Uso *Off-Label*, *Off-Label*, Estudos de Coortes.

## Abstract

**Objective:**

to investigate sociodemographic and clinical characteristics of users of atypical antipsychotics receiving care via the Specialized Component of Pharmaceutical Assistance (*Componente Especializado da Assistência Farmacêutica* - CEAF), for the treatment of schizophrenia in Brazil, between 2008 and 2017.

**Methods:**

this was a retrospective cohort study using records of the authorizations for high complexity procedures retrieved from the Outpatient Information System of the Brazilian National Health System, from all Brazilian states.

**Results:**

of the 759,654 users, 50.5% were female, from the Southeast region (60.2%), diagnosed with paranoid schizophrenia (77.6%); it could be seen a higher prevalence of the use of risperidone (63.3%) among children/adolescents; olanzapine (34.0%) in adults; and quetiapine (47.4%) in older adults; about 40% of children/adolescents were in off-label use of antipsychotics according to age; adherence to CEAF was high (82%), and abandonment within six months was 24%.

**Conclusion:**

the findings expand knowledge about the sociodemographic and clinical profile of users and highlight the practice of off-label use.

## Resumo

**Objetivo:**

investigar características sociodemográficas e clínicas de usuários de antipsicóticos atípicos assistidos pelo Componente Especializado da Assistência Farmacêutica (CEAF), para tratamento da esquizofrenia no Brasil, de 2008 a 2017.

**Métodos:**

estudo de coorte retrospectivo utilizando registros das autorizações de procedimentos de alta complexidade do Sistema de Informações Ambulatoriais do Sistema Único de Saúde, de todos os estados brasileiros.

**Resultados:**

dos 759.654 usuários, 50,5% eram do sexo feminino, da região Sudeste (60,2%), diagnosticados com esquizofrenia paranoide (77,6%); observou-se maior prevalência de uso da risperidona (63,3%) entre crianças/adolescentes; de olanzapina (34,0%), em adultos; e quetiapina (47,4%), nos idosos; cerca de 40% das crianças/adolescentes estavam sob uso *off-label* de antipsicóticos segundo a idade; a adesão ao CEAF foi alta (82%), e o abandono em seis meses foi de 24%.

**Conclusão:**

os achados ampliam o conhecimento sobre perfil sociodemográfico e clínico dos usuários e destacam a prática do uso *off-label*.

## Resumen

**Objetivo:**

investigar las características sociodemográficas y clínicas de los usuarios de antipsicóticos atípicos, atendidos por el Componente Especializado de Asistencia Farmacéutica (CEAF) para el tratamiento de la esquizofrenia en Brasil, de 2008 a 2017.

**Métodos:**

estudio de cohorte retrospectivo utilizando registros de autorizaciones de trámites de alta complejidad del Sistema de Información Ambulatorio del SUS, de todos los estados brasileños.

**Resultados:**

de los 759.654 usuários identificados, el 50,5% era del sexo feminino de la región Sudeste (60,2%), diagnosticadas con esquizofrenia paranoide (77,6%). Hubo una mayor prevalencia de risperidona (63,3%) entre niños y adolescentes; de olanzapina (34,0%) en adultos; y quetiapina (47,4%) en ancianos. Alrededor del 40% de los niños/adolescentes estaba bajo uso no autorizado de antipsicóticos según la edad. La adherencia al CEAF fue alta (82%), y la deserción a los seis meses fue del 24%.

**Conclusión:**

los hallazgos amplían el conocimiento sobre el perfil sociodemográfico y clínico de los usuarios y destacan la práctica del uso off-label.

**Table:** 

Study contributions	
Main results	The pattern of use of atypical antipsychotics for the treatment of schizophrenia via the SUS differed according to age group. A significant proportion of children and adolescents were exposed to off-label use.
Implications for services	The frequencies of off-label use, adherence to, and abandonment of the pharmaceutical assistance program provide useful information for planning and management, as well as possible improvement in the care of these users.
Perspectives	The findings reinforce the importance of improving the care provided to users of atypical antipsychotics, especially those in the extremes of age group. Further studies on off-label use and the reasons for the abandonment of the program are needed.

## INTRODUCTION

Schizophrenia is a severe, chronic, debilitating mental disorder that affects about 0.7% of the world’s population.^1^ Although its prevalence is relatively low, the disease has shown an increasing trend, especially in low- and middle-income countries.^2^ In Brazil, the prevalence of psychoses in general ranges from 0.3 to 2.4%; and of schizophrenia is close to 1% of the population.^3^ ^),(^ ^4^

The onset of symptoms of schizophrenia occurs in young adults, usually before the age of 25 in men and before 35 years old in women.^1^ Early-onset schizophrenia (before 18 years old) and late-onset schizophrenia (after 40 years old) are not very common, diagnosis is more complex, and evidence on effectiveness and safety of treatment is limited.^5^ ^),(^ ^6^

Antipsychotic medications play a central role in the treatment of schizophrenia and other psychotic disorders and, since they were introduced on the market in the 1990s, they have revolutionized schizophrenia treatment, especially in cases of refractoriness.^7^ A systematic review that included 25 clinical trials, most of them conducted in the United States, showed that there are no differences in effectiveness among atypical antipsychotics, except clozapine.^8^ The differences lie in the adverse effect profile, whose main advantage over conventional antipsychotics, is a higher tolerability and lower risk of extrapyramidal effects.^7^ However, these drugs have a higher risk of metabolic syndrome, and a higher cost.^9^

The Brazilian National Health System (*Sistema Único de Saúde* - SUS) provides both conventional (chlorpromazine and haloperidol) and atypical antipsychotics (clozapine, olanzapine, quetiapine, risperidone and ziprasidone) for the treatment of schizophrenia and schizoaffective disorder.^10^ ^),(^ ^11^ Atypical antipsychotics are considered high-cost medications, belong to the pharmaceutical assistance program called Specialized Component of Pharmaceutical Assistance (*Componente Especializado da Assistência Farmacêutica* - CEAF), and are dispensed only after analysis of the request and compliance with the requirements described in the specific Brazilian clinical protocol.^12^

In recent years, there has been a marked increase in the use of atypical antipsychotics in several parts of the world (The United States, Europe, Germany, Taiwan), in the general population and in more vulnerable groups, such as children and older adults.^13^ ^),(^ ^14^ The main concerns with regard to this problem include widespread off-label use (unlicensed use, in disagreement with the information provided in the package leaflet regarding the indication, dose, age or routes of administration)^15^ and the exposure of the most vulnerable groups to potential adverse effects caused by these medications.

The costs of atypical antipsychotics are high and accounted for the largest share of expenditures related to the treatment of schizophrenia via SUS.^16^ In the period from 2000 to 2010, olanzapine accounted for about 63% of total expenditure on atypical antipsychotics, and clozapine users had the highest average expenditure on outpatient psychiatric follow-up and psychiatric hospitalization.^16^

Taking into consideration the significant consumption of atypical antipsychotics worldwide, the exposure to the risks associated with their use^13^ ^),(^ ^14^ and the high cost this represents, particularly for the Brazilian health system,^16^ it is both timely and justifiable to know the profile of the use of these medications in the Brazilian real-life scenario.

The objective of this study was to investigate the sociodemographic and clinical characteristics of users of atypical antipsychotics receiving care from the CEAF, for the treatment of schizophrenia.

## METHODS

This was a retrospective cohort study, based on data from the SUS Outpatient Information System (*Sistema de Informações Ambulatoriais do Sistema Único de Saúde* - SIA/SUS). The records related to Authorizations for High Complexity Procedures (APACs) in all Brazilian Federation Units, stored in the database of the access to medicine program belonging to CEAF of SIA/SUS, were used as a source of information.^12^

Data were obtained from the Brazilian National Health System Information Technology Department (*Departamento de Informática do Sistema Único de Saúde* - DATASUS), at <www.datasus.gov>, in April 2018.

This administrative database provides information on the supply of medicines belonging to CEAF, formerly called high-cost medicines, at the outpatient level, for the treatment of certain diseases, according to the Clinical Protocols and Therapeutic Guidelines (*Protocolos Clínicos e Diretrizes Terapêuticas* - PCDT).^12^

Access to these medications occurs when the prescribing physician fills out an Appraisal for Request Evaluation, and Authorization of Medication (*Laudo de solicitação de medicamentos* - LME). Subsequently, it is analyzed and checked whether the LME complies with the requirements present in the PCDT. If approved, the LME is authorized; then, an APAC, related to the supply of medicines, is generated with user registration on the national database for management and billing purposes.^12^ This process ensures the supply of medicines for up to three months (90 days). After database entry, if there is a need to continue the treatment, the whole process is repeated, a new APAC record is generated and inserted into the database.

CEAF provides the following oral atypical antipsychotics for the treatment of schizophrenia and schizoaffective disorder: clozapine, olanzapine, risperidone, quetiapine and ziprasidone.^10^ ^),(^ ^11^ For the treatment of schizophrenia or schizoaffective disorder, PCDT recommends the use of these atypical antipsychotics as monotherapy, with no order of preference, except for clozapine. The choice should be made according to the users safety and tolerability profile. In case of therapeutic failure (defined as: the use of any of these medications for at least six weeks, at recommended doses, without improving by at least 30% in the Brief Psychiatric Rating Scale) or in case of intolerance due to adverse effects, a second attempt should be made, with another antipsychotic. Clozapine is reserved for cases of refractoriness to at least two other antipsychotics used for at least six weeks at recommended doses, and if there is no improvement of at least 30% in the Brief Psychiatric Rating Scale, or if there is a high risk of suicide or tardive dyskinesia of significant consequence.^10^ ^),(^ ^11^ The PCDT does not provide guidance on the specifications for treatment in children, adolescents and older adults.

All users of atypical antipsychotics diagnosed with schizophrenia and schizoaffective disorder admitted at least once to the access to medicine program belonging to CEAF between January 1, 2008 and December 31, 2017, were included. None of the users identified in this period were excluded.

The start date of this study, 2008, is justified by the fact that individualized secondary data are publicly available (unrestricted access) in that database only from that year onwards. Users may have had more records as of 2018; however, the period selected by these researchers was from 2008 to 2017 and therefore, the records from 2018 or later were not included in this study basis.

The data of all users identified and analyzed were retrieved from the APAC records provided by the SIA/SUS. These records were compared by the number of the National Health Card (*Cartão Nacional de Saúde* - CNS), and by deterministic matching, in order to identify possible multiple records of the same user. The first date of dispensing an atypical antipsychotic identified on the SIA/SUS was considered as the date of inclusion in the study. There was no exclusion of any users identified on the database. It is noteworthy that SIA/SUS makes available the encrypted data, and with these data the deterministic matching was carried out, and also taking into consideration, sex, age and location where the APAC was authorized.

The following categories of variables were considered:

a) Demographics
- sex (female; male);
- age group (in full years: up to 10; 11 to 18; 19 to 29; 30 to 19; 40 to 49; 50 to 59; 60 to 69; 70 to 79; 80 to 89; 90 to 99);
- average age;
- race/skin color, self-reported (White; Black; mixed race; Asian; Indigenous; unidentified);
- national region of residence (South; Southeast; Midwest; North; Northeast); and
- year of inclusion in the CEAF program, i.e. (i) the date when the individual received the first atypical antipsychotic via the program or (ii) the date of the first dispensing, in the period from January 2008 to December 2017).
b) Clinical
- principal diagnosis, according to the International Statistical Classification of Diseases and Related Health Problems (ICD-10), specifically its items related to schizophrenia, F20.0 to F20.8 and F25.0 to F25.2;
- average treatment time (number of months registered on SIA/SUS), atypical antipsychotic used when entering the CEAF program (clozapine, olanzapine, risperidone, quetiapine and ziprasidone) and have switched the atypical antipsychotic at least once (yes; no).
d) Off-label use

The classification of off-label use^15^ of atypical antipsychotics, according to indication for use and age, was made by consulting the information contained in the package leaflets approved by the Brazilian Health Regulatory Agency (*Agência Nacional de Vigilância Sanitária* - ANVISA), available in the Electronic Package Leaflet database.^17^ According to ANVISA, atypical antipsychotics are approved for the treatment of schizophrenia as of the following ages: clozapine (> 18 years old), olanzapine (> 13 years old), risperidone (> 13 years old), quetiapine (> 13 years old) and ziprasidone (> 18 years old).^15^

e) Adherence to the pharmaceutical assistance program - CEAF

Adherence to the program is measured by the proportion of time in which the user is in possession of the medication, over the total duration of the study time. It was calculated by the total number of days covered by the prescription during the study (or final date of submission of the last APAC, subtracted by the starting date of the first APAC), divided by the study time (date of the final study period, December 2017, subtracted from the starting date of the first APAC) and multiplied by 100. This proportion of time was categorized as follows: adherent, ≥ 80%; partially adherent, 50-79%; non-adherent, < 50%. This adherence measure was the result of an adaptation, based on a methodology developed by another researcher.^18^ ^),(^ ^19^

f) Abandonment of the pharmaceutical assistance program - CEAF

This is the proportion of users who leave the program before a six-month follow-up. We evaluated whether the date of the last replenishment occurred before completing a six- month follow-up or whether the case presented only one entry in the APAC database. This measure of program abandonment is an adaptation of a methodology developed by another researcher.^18^ ^),(^ ^19^

Demographic and clinical characteristics were distributed among three age groups: children and adolescents (up to 18 years old); adults (19 to 59 years old); and older adults (≥ 60 years). The continuous variables were expressed as mean and standard deviation, and categorical variables as percentage, for descriptive statistics. All data were analyzed using the Stata software, version 14.2.

The data used in this study were retrieved from DATASUS, a public domain database, which does not provide specific information that can identify users, therefore, it was not submitted to and analyzed by a Research Ethics Committee.

## RESULTS

A total of 759,654 users of atypical antipsychotics, diagnosed with schizophrenia (F20.0 to F20.8) or schizoaffective disorder (F25.0 to F25.2), with at least one entry in the SIA/SUS database, were identified. Most of these users were adults (19 to 59 years old: n = 522,071, 68.7%), followed by the elderly (≥ 60 years old: n = 168,999, 22.2%) and children/adolescents (up to 18 years old: n = 68,584, 9.1%).

Overall, 50.5% were adult female, with an average age of 44 years (± 0.1), living in the Southeast region of the country. The highest proportion of children/adolescents (n = 47,042, 68.6%) and adults (n = 266,168, 50,9%) were male, while the majority of the elderly were female (n = 106,583, 63.1%) (Table 1).

**Table 1:** Demographic characteristics of users who received atypical antipsychotics via the Brazilian National Health System, their national region of residence and year of entry into the database, Brazil, 2008-2017

Variables	Children and adolescents (up to 18 years old)	Adults (19 to 59 years old)	Older adults (60 years and older)	Total
	N = 68,584 (%)	N = 522,071 (%)	N = 168,999 (%)	N = 759,654 (%)
Sex				
Female	21,542 (31.4)	255,903 (49.1)	106,583 (63.1)	384,028 (50.5)
Male	47,042 (68.6)	266,168 (50.9)	62,416 (36.9)	375,626 (49.5)
Age group (full years)				
≤ 10	16,344 (23.8)			16,344 (2.2)
11-18	52,240 (76.2)			52,240 (6.9)
19-29		133,714 (25.6)		133,714 (17.6)
30-39		146,518 (28.1)		146,518 (19.3)
40-49		137,630 (26.4)		137,630 (18.2)
50-59		104,209 (19.9)		104,209 (13.7)
60-69			60,524 (35.8)	60,524 (7.9)
70-79			54,794 (32.4)	54,794 (7.2)
80-89			45,138 (26.7)	45,138 (5.9)
90-99			8,543 (5.1)	8,543 (1.1)
Race/skin color (self-reported)				
White	8,241 (12.0)	55,274 (10.6)	24,641 (14.6)	88,156 (11.1)
Black	773 (1.1)	5,809 (1.1)	1,430 (0.8)	8,012 (1.0)
Mixed race	5,620 (8.2)	34,782 (6.6)	8,612(5.1)	49,014 (6.2)
Asian	1,536 (2.2)	12,045 (2.3)	2,783 (1.6)	16,364 (2.1)
Indigenous	33 (0.1)	93 (0.1)	20 (0.1)	146 (0.1)
No information	52,381 (76.4)	414,068 (79.3)	131,513 (77.8)	633,962 (79.5)
National region of residence				
North	1,855 (2.7)	14,313 (2.7)	2,067 (1.2)	18,235 (2.4)
Northeast	14,312 (20.8)	104,933 (20.1)	23,416 (13.9)	142,661 (18.8)
Southeast	39,681 (57.9)	296,424 (56.8)	121,524 (71.9)	457,629 (60.3)
South	8,829(12.9)	66,253 (12.7)	13,133 (7.7)	88,215 (11.6)
Midwest	3,907 (5.7)	40,148 (7.7)	8,859 (5.3)	52,914 (6.9)
Year of entry into the pharmaceutical assistance program - CEAF^a^				
2008	12,168 (17.7)	118,427 (22.7)	24,640 (14.6)	155,235 (20.4)
2009	6,116 (8.9)	46,750 (8.9)	13,191 (7.8)	66,057 (8.7)
2010	6,321 (9.2)	43,510 (8.3)	16,141 (9.6)	65,972 (8.7)
2011	6,545 (9.5)	44,545 (8.5)	15,087 (8.9)	66,177 (8.7)
2012	5,798 (8.5)	37,571 (7.2)	12,960 (7.7)	56,329 (7.4)
2013	5,864 (8.6)	41,059 (7.9)	14,600 (8.6)	61,523 (8.1)
2014	6,169 (9.0)	41,162 (7.9)	16,056 (9.5)	63,387 (8.4)
2015	6,991 (10.2)	53,317 (10.2)	19,589 (11.6)	79,897 (10.5)
2016	6,717 (9.8)	53,678 (10.3)	19,499 (11.5)	79,894 (10.5)
2017	5,895 (8.6)	42,052 (8.1)	17,236 (10.2)	65,183 (8.6)

a) CEAF: Specialized Component of Pharmaceutical Assistance/Department of Pharmaceutical Assistance, Secretariat of Science and Technology and Strategic Inputs, Ministry of Health.

The most common diagnosis, regardless of age group, was paranoid schizophrenia (F20.0; n = 589,718, 77.6%), followed by other types of schizophrenia (F20.8; n = 83,530, 11.0%). The mean treatment time was 32 months (± 0.4), ranging from 24.7 (± 0.1) months for the elderly, 28.8 (± 0.1) months for children/adolescents and 34.9 (± 0.1) months for adults. The most prescribed atypical antipsychotic over the ten years evaluated was risperidone (n = 251,352, 33.1%), followed by olanzapine (n = 224,861, 29.6%), quetiapine (n = 210,412, 27.7%), ziprasidone (n = 38,543, 5.1%) and clozapine (n = 34,486, 4.5%). When taking into consideration only children/adolescents, risperidone stands out with 63.3%. Olanzapine was the most prescribed antipsychotic to adults (n = 177,787, 34.1%), followed by risperidone (n = 158,415, 30.3%). Among the elderly, there was a predominance of the use of quetiapine (n = 80,139, 47.4%), followed by risperidone (n = 49,512, 29.3%). Approximately 13.5% (n = 9,242) of children/adolescents, 14,6% (n = 76,210) of adults and 8,6% (n = 14,632) of the elderly switched their atypical antipsychotic to another, at least once, during treatment (Table 2).

**Table 2:** Clinical characteristics of users who received atypical antipsychotics via the Brazilian National Health System, Brazil, 2008-2017

Variables	Children and adolescents (up to 18 years old)	Adults (19 to 59 years old)	Older adults (60 years and older)	Total
	N = 68,584 (%)	N = 522,071 (%)	N = 168,999 (%)	N = 759,654 (%)
Principal diagnosis				
Paranoid schizophrenia	49,847 (72.7)	413,848 (79.3)	126,023 (74.6)	589,718 (77.6)
Hebephrenic schizophrenia	3,462 (5.1)	15,081 (2.9)	3,372 (2.0)	21,915 (2.9)
Catatonic schizophrenia	306 (0.4)	2,346 (0.4)	674 (0.4)	3,326 (0.4)
Undifferentiated schizophrenia	2,021 (2.9)	12,961 (2.5)	4,093 (2.4)	19,075 (2.5)
Post-schizophrenic depression	203 (0.3)	2,667 (0.5)	1,198 (0.7)	4,068 (0.5)
Residual schizophrenia	858 (1.3)	16,071 (3.1)	5,794 (3.4)	22,723 (3.0)
Simple schizophrenia	944 (1.4)	5,304 (1.0)	2,021 (1.2)	269 (1.1)
Other types of schizophrenia	10,499 (15.3)	48,758 (9.4)	24,273 (14.4)	83,530 (11.0)
Bipolar schizoaffective disorder	202 (0.3)	1,788 (0.3)	629 (0.4)	2,619 (0.3)
Schizoaffective disorder, depressive type	82 (0.1)	1,764 (0.3)	500 (0.3)	2,346 (0.4)
Schizoaffective disorder, mixed type	160 (0.2)	1,483 (0.3)	422 (0.2)	2,065 (0.3)
Atypical antipsychotic used when entering the pharmaceutical assistance program - CEAF^a^				
Clozapine	1,518 (2.2)	30,311 (5.8)	2,657 (1.6)	34,486 (4.5)
Olanzapine	13,380 (19.5)	177,787 (34.1)	33,694 (19.9)	224,861 (29.6)
Risperidone	43,425 (63.3)	158,415 (30.3)	49,512 (29.3)	251,352 (33.1)
Quetiapine	8,106 (11.8)	122,167 (23.4)	80,139 (47.4)	210,412 (27.7)
Ziprasidone	2,155 (3.2)	33,391 (6.4)	2,997 (1.8)	38,543 (5.1)
Users who switched the atypical antipsychotic medication at least once				
Yes	9,242 (13.5)	76,210 (14.6)	14,632 (8.6)	100,084 (13.2)
No	59,342 (86.5)	445,861 (85.4)	154,367 (91.4)	659,570 (86.8)

a) CEAF: Specialized Component of Pharmaceutical Assistance/Department of Pharmaceutical Assistance, Secretariat of Science and Technology and Strategic Inputs, Ministry of Health.

**Figure 1:**
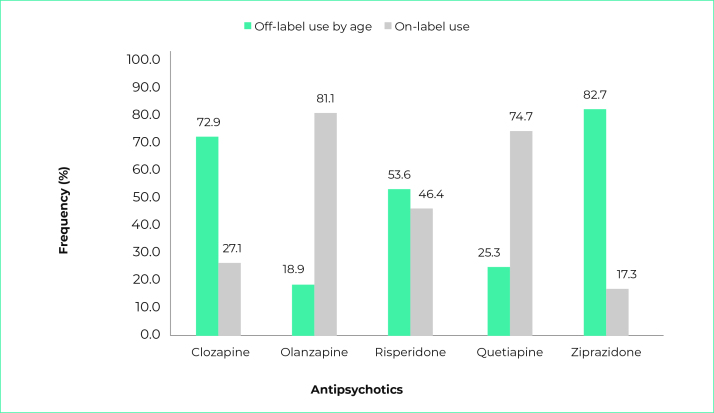
Frequency of children and adolescents who received atypical antipsychotics (N = 30,755) according to the type of use (on-label or off-label), Brazil, 2008-2017

Overall, 30,755 (44.8%) of children and adolescents were in off-label use. Among users under the age of 13, the off-label use of risperidone (53.6%), olanzapine (18.9%) and quetiapine (25.3%) stood out. Among those under 18 years of age, off-label use of ziprasidone (82.7%) and clozapine (72.9%) was predominant (Figure 1).

Of the users identified in this study, 24.8% abandoned the program within the first six months and 8.2% had only one entry in the database, i.e., the atypical antipsychotic was obtained only once. The frequency of adherence to the pharmaceutical assistance program or the atypical antipsychotic replenishment during the study was high: 81.9% of the users were adherent to the CEAF program and remained in the database for more than 80% of the study period (Table 3).

**Table 3:** Adherence and abandonment of users of atypical antipsychotics receiving care via the pharmaceutical assistance program of CEAF,^a^ Brazil, 2008-2017

Variables	N = 759,654 (100.0%)
Frequency of user adherence to replenishment of atypical antipsychotics	
≥ 80%	622,191 (81.9)
50-79%	81,903 (10.8)
< 50%	55,561 (7.3)
Frequency of users who abandoned the replenishment of atypical antipsychotics	
User with a single database entry	62,550 (8.2)
Users who left the program before six months	188,786 (24.8)
Users who have not abandoned the program before six months	508,318 (67.0)

a) CEAF: Specialized Component of Pharmaceutical Assistance/Department of Pharmaceutical Assistance, Secretariat of Science and Technology and Strategic Inputs, Ministry of Health.

## DISCUSSION

The findings of this study provide information on the sociodemographic and clinical characteristics of users of atypical antipsychotics diagnosed with schizophrenia or schizoaffective disorder, receiving care via the SUS, in different age groups. The pattern of use differed according to age group: risperidone was the most prescribed atypical antipsychotic for children and adolescents, olanzapine for adults and quetiapine for the elderly. A significant proportion of children and adolescents were exposed to off-label use of antipsychotics.

The program abandonment rate exceeded 20% in the first six months of use. Adherence and abandonment rate of the pharmaceutical assistance program, calculated according to the time the medication is in the possession of the individual, provides useful information for planning and managing pharmaceutical assistance.

The highest proportion of males among adults and children/adolescents corroborates the findings of other studies and suggests, once again, that schizophrenia is more prevalent among males, and earlier in boys.^1^ ^),(^ ^13^ ^),(^ ^20^ However, there was a higher proportion of females among the elderly, consistent with other findings of a Taiwanese study.^14^

Over the ten years of this study, the majority of users, who were identified, lived in the Southeast region when they entered the program, a finding that may suggest a relationship between socioeconomic factors and the onset of schizophrenia. Southeastern Brazil is the region with the highest human development index (HDI: 0.79), the highest household income *per capita* and the greatest access to health services, however with a high level of social inequality.^21^ A national survey reaffirmed the present findings and that schizophrenia is more prevalent in the Southeast region than in other Brazilian regions, which can be attributed to the way of life of the population in this region.^22^

Differences in the profile of atypical antipsychotics use were observed according to age group. Children are potentially more susceptible to adverse effects of antipsychotic medications and the choice of antipsychotics should be made according to the metabolic, cardiovascular and hormonal profile of the child.^23^ Over the ten years studied, risperidone was the most commonly used atypical antipsychotic among children and adolescents, a pattern found in studies conducted in Taiwan, Germany and Brazil.^13^ ^),(^ ^20^ ^),(^ ^24^

Approximately 9% of the identified users were aged 18 years or younger and most of these medications are not recommended for this age group, because their effectiveness and safety have not been established yet. The ANVISA and the U.S. Food and Drug Administration (FDA), have approved the use of risperidone, olanzapine and quetiapine for treating schizophrenia only among those aged 13 years and older; and clozapine and ziprasidone, in those aged 18 years and older.^17^ ^),(^ ^25^ The significant age-related off-label use in children and adolescents in this study was also found in other studies conducted in Denmark and Brazil, and represents a major concern for health authorities.^15^ ^),(^ ^20^ ^),(^ ^26^ Although it is common knowledge that off-label use of medications can lead to more serious adverse events, and occur more frequently, it is a clinically accepted practice in pediatrics worldwide.^15^

Olanzapine was the most commonly used atypical antipsychotic among adults, followed by risperidone, quetiapine, ziprasidone and clozapine. The trend in the use of olanzapine corroborates the findings of another cohort study conducted in Brazil, from 2000 to 2010.^16^ Systematic reviews have shown that, olanzapine causes more weight gain and metabolic disorders, when compared to other atypical antipsychotics ^(^ ^27^ which may lead to health problems for users, low adherence, and even determine its switching to another medication that presents less risk of causing weight gain.^28^

Quetiapine was the most commonly used atypical antipsychotic among the elderly, followed by risperidone. A systematic review showed that this medication presents lower propensity for movement disorders than risperidone, but causes more dizziness, dry mouth and drowsiness.^29^ Older adults have benefits related to atypical antipsychotics, which are well tolerated in this age group as long as their use poses low risk for extrapyramidal effects, metabolic disorders and weight gain.^6^ However, physiological changes due to advanced age may result in prolongation of effects of antipsychotics and greater susceptibility to adverse effects, therefore, they should be used in this age group with caution and in low doses.^6^

Despite its superiority over other atypical antipsychotics, clozapine was the least prescribed antipsychotic medication in all age groups. This can be explained by the fact that PCDT recommends its use only after therapeutic failure of two other antipsychotics.^10^ ^),(^ ^11^

According to APAC records, the findings showed that most users adhered to the antipsychotic dispensing or replenishment program. Further analyses should be conducted in order to assess the prevalence of gaps in replenishment and investigate risk factors for treatment discontinuation. Approximately 33% of users abandoned the program or stopped antipsychotics before six months, a fact that may boost future research on the causes of abandonment and its consequences for the health care system. Further research is needed to assess users adherence and abandonment, as well as the profile of those who abandon treatment early.

The strength of this study certainly lies in the fact that it is one of the few studies aimed at investigating the use of atypical antipsychotics in individuals with schizophrenia or schizoaffective disorder via the SUS, without age restriction, over a ten-year period. We used an important and large SUS database, the SIA/SUS, which is publicly accessible, but little explored in Brazilian research due to its difficult management, which requires data scientist and epidemiologist knowledge.

The main limitations of this study are related to the database. It is a large administrative database, with records to be reported to SUS, and not a database developed for clinical research purposes. Therefore, these data are subject to data feed errors, such as the filling in of erroneous data related to sex, age, national region of residence, ICD-10 and other fields in the APAC, errors that may underestimate or overestimate the findings related to the user profile. However, the data were double-checked in order to increase its reliability. Some non-mandatory fields of APAC were not filled in, such as race/skin color, presence of comorbidities, mortality, reasons for switching antipsychotics, for non-adherence or abandonment of the dispensing program, which limited certain analyses. These findings represent important gaps in knowledge, which can motivate new research and even points to be improved in this relevant database. It is noteworthy that the methodology used to estimate the adherence was theoretical, based on the antipsychotic dispensing documents and, therefore, may not represent the true behavior of treatment adherence.

The users evaluated in this study are those who obtain their medications via the SUS, and, therefore, there is no information about the pattern of antipsychotics use of users who obtained these medications from the private healthcare sector. It is worth emphasizing that the diagnosis of schizophrenia and schizoaffective disorder provided in the database, was used.

It can be concluded that the data broadened the knowledge about the sociodemographic and clinical profile of users of atypical antipsychotics receiving care via the SUS. A considerable number of users belonging to extremes of age group - children and the elderly - were exposed to the effects of the use of these medications, and deserve more attention. The finding of off-label use, according to age, points to the inappropriate use of these medications. The development of specific clinical protocols for the treatment of schizophrenia in children/adolescents and in the elderly could help professionals in clinical practice, optimize treatment and the quality of care provided by SUS, especially to the most vulnerable individuals.

The use of large administrative databases in the public domain, such as the records of the authorization for high complexity procedures (*autorização de procedimentos de alta complexidade* - APAC) allows the generation of data of interest to public health. Decision-making guided by real-life data is a promising trend that can contribute more assertively to the planning and management of actions, the supply of high-value medication, the rational use of resources and medicines, and the improvement of the treatment and follow-up of schizophrenia within the SUS.
